# Solitary mesenteric vascular anomaly presenting as acute abdomen

**DOI:** 10.4103/0971-9261.43820

**Published:** 2008

**Authors:** C. R. Thambidorai, A. Wahab, A. H. Hamzaini

**Affiliations:** Paediatric Surgery Unit, Departments of Surgery and ^1^Radiology, University Kebangsaan of Malaysia, Cheras, 56000 Kuala Lumpur, Malaysia

**Keywords:** Child, hemangioma, mesentery, vascular malformation, solitary vascular anomaly

## Abstract

A 4-year-old girl with a solitary vascular anomaly of the mesentery presented with acute lower abdominal pain. Despite the use of ultrasound, computed tomography scan and image-guided core biopsies, the lesion was initially mistaken for an inflammatory intra-abdominal mass. The correct diagnosis was made at laparotomy. Solitary vascular anomaly of the mesentery is rare and its presentation as an acute abdomen has not been reported before.

## INTRODUCTION

Vascular anomalies have been classified by the International Society for the study of vascular anomalies into hemangiomas and vascular malformations (VM).[1] Hemangiomas may present at birth or soon after birth, proliferate during infancy and involute during childhood (usually up to 12 years.). VMs may be present at birth or develop later; they do not involute but tend to increase in size as the child grows.[1,2]

Solitary vascular anomaly of the mesentery of the small bowel is extremely rare. Only two cases of solitary abdominal hemangioma in children have been described in the literature.[3,4]

## CASE REPORT

A 4-year-old Malay female was seen at a private center with abdominal pain, vomiting and fever of five days duration. Laparotomy was done through a Lanz incision with a provisional diagnosis of acute appendicitis. This revealed a firm mass near the right kidney. The mass was presumed to be either a Wilms’ tumor or a retroperitoneal neoplasm. The wound was closed, and she was referred to our center for further management.

**Figure 1:**
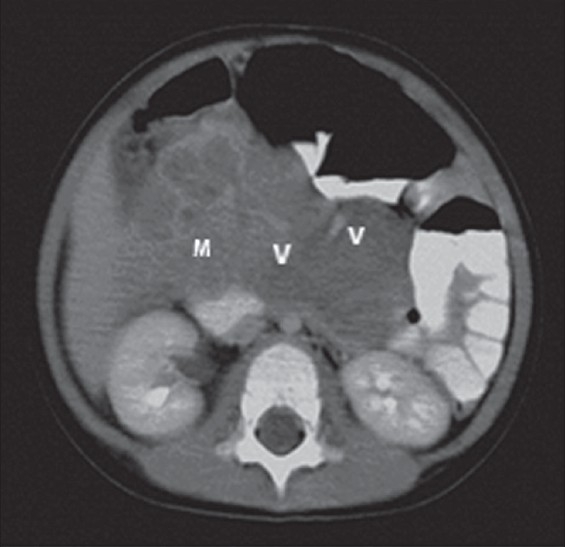
The first contrast-enhanced computed tomography (CECT) scan of the abdomen shows the heterogeneously enhancing mass (M) anterior to the aorta and inferior vena cava. The superior mesenteric vessels (V) are displaced anteriorly

On arrival, she was febrile but the abdominal mass could not be palpated, possibly due to the recent laparotomy. Full blood count revealed leukocytosis of 22 × 10^9^/L with predominant neutrophilia, hemoglobin of 10 g/dl and platelet count of 400 × 10^12^/L. Alpha-fetoprotein and β-human chorionic gonadotropin levels were normal. Abdominal ultrasound (US) showed a mass with mixed echoes in relation to the lower pole of the right kidney. Contrast-enhanced computed tomography (CECT) revealed a mass with a size of 14 × 7 × 11 cm with cystic spaces within it, extending superiorly to the level of celiac artery, inferiorly down to the pelvic cavity and laterally to the splenic hilum [Figure 1]. There was no clear fat plane between the mass and the pancreas. Both kidneys were normal. The celiac and superior mesenteric vessels were stretched by the mass. Contrast injection revealed heterogeneous enhancement of the mass. Infective mesenteric lymphadenitis, inflammatory myofibroblastic tumor (IMT), abdominal lymphoma, and peripheral neuroectodermal tumor (PNET) were considered in the differential diagnoses based on the CECT findings. She was started on amoxycillin / clavulanic acid therapy.

US-guided core biopsies showed strips of smooth muscle tissue interspersed with collagen. Lymphocytes, plasma cells, neutrophils and hemosiderin-laden macrophages were also present. Chronic inflammatory mass of infective origin and IMT were considered in the differential diagnosis based on histology.

**Figure 2:**
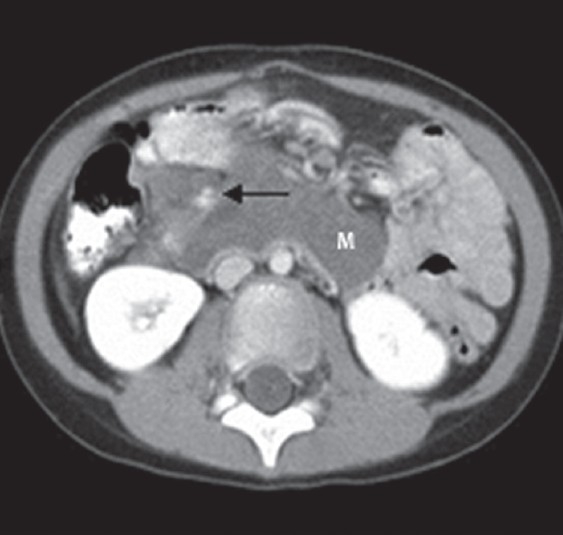
The second CECT scan of the abdomen shows that the mass (M) has significantly reduced in size compared to the first scan. The horizontal black arrow points to the superior mesenteric vessels

Meanwhile, she improved with resolution of fever and the abdominal pain. US and CECT scans repeated three weeks later showed the size of the original mass had decreased to 7.5 × 2.8 × 5.5 cm [Figure 2]. Her leukocyte count decreased to 6.5 × 10^9^/L. Based on the initial presentation as an intra-abdominal infection, the subsequent reduction in the mesenteric mass and the core biopsy findings, IMT was considered the most likely. The oral antibiotics were continued for a total period of six weeks.

A repeat CECT scan at eight weeks, however, showed persistence of the retroperitoneal mass of same size as the second scan. Diagnostic laparoscopy revealed a mass within the mesenteric root that extended to the pancreas above. The limits of the mass were not seen clearly through the mesentery. The surface of the mass showed dilated vessels, suggesting a diagnosis of a vascular anomaly or a highly vascular tumor. At laparotomy, the mass was found to be an isolated vascular anomaly in the mesentery. It measured about 8 × 5 × 5 cm and predominantly consisted of thin-walled vessels with slow filling on compression. The adjacent bowel was not involved by the mass. The vascular lesion was excised completely with ligation of the small feeding vessels from the mesenteric vessels. The postoperative period was uneventful.

Histology revealed numerous flattened endothelium-lined vascular spaces of varying sizes with areas of focal thrombosis. Scattered lymphatic follicles with germinal centers were seen within the vessel walls and the surrounding stroma. The diagnosis of a solitary vascular anomaly of the mesentery that could represent either a vascular malformation with low flow or an involuting hemangioma was made.

## DISCUSSION

Mesenteric vascular anomalies may present with gastrointestinal or intraperitoneal hemorrhage and rarely with hepatic dysfunction and consumptive coagulopathy.[3,4] Intralesional hemorrhage, thrombosis, secondary infection and traction or torsion of the mesentery may result in presentation as an acute abdomen. Preoperative diagnosis of the vascular anomalies of the mesentery is difficult. Small biopsies obtained preoperatively may be inconclusive because of secondary changes such as inflammatory cell infiltration, myofibroblastic proliferation and xanthogranulomatous inflammation.[2–5] Lymphangioma, IMT, lymphoma, fibrosarcoma and cystic mesothelioma simulate vascular anomalies in the mesentery on histology and imaging.[5,6] GLUT-1 is a newly described, highly specific immunohistochemical marker for infantile hemangioma in the proliferative phases and is useful in deep-seated lesions where the biopsies obtained for diagnosis may be small.[7]

In conclusion, a solitary vascular anomaly of the mesentery should be considered in the differential diagnosis of an obscure abdominal mass in a child even when the presentation is as an acute abdomen.
